# Curcumin supplementation reduces blood glucose and serum lipids of Brazilian women with high waist circumference: a randomized clinical trial

**DOI:** 10.20945/2359-3997000000513

**Published:** 2022-09-20

**Authors:** Pamela Cristina de Sousa Guardiano Reis, Ana Gabriella Pereira Alves, Lídia Andreu Guillo, Menandes Alves de Sousa, Neidiane Rosa Trindade, Maria Sebastiana Silva

**Affiliations:** 1 Universidade Federal de Goiás Faculdade de Educação Física e Dança (FEFD) Laboratório de Fisiologia, Nutrição e Saúde (LAFINS) Goiânia Goiás Brasil Laboratório de Fisiologia, Nutrição e Saúde (LAFINS), Faculdade de Educação Física e Dança (FEFD), Universidade Federal de Goiás (UFG) – Campus Samambaia, Goiânia, Goiás, Brasil; 2 Universidade Federal de Goiás Instituto de Ciências Biológicas Departamento de Bioquímica e Biologia Molecular Goiânia Goiás Brasil Instituto de Ciências Biológicas, Departamento de Bioquímica e Biologia Molecular, Universidade Federal de Goiás (UFG) – Campus Samambaia, Goiânia, Goiás, Brasil

**Keywords:** Curcumin, women, blood glucose, cholesterol, triglycerides

## Abstract

**Objective:**

To evaluate the effect of curcumin supplementation on the body compositions and biochemical parameters of Brazilian women with high waist circumferences.

**Materials and methods:**

This is a blind, randomized, placebo-controlled clinical trial conducted in 2016 with 35 Brazilian women with high waist circumference (WC). In total, 80 participants were randomized [38 in the placebo group (PG) and 42 in the supplemented group (SG)], but at the end of the protocol, 20 individuals in the PG and 15 in the SG were evaluated. The sample consumed one capsule of curcumin (500 mg/day) (Curcumin C3 Complex^®^) or a placebo for 90 days. Body weight, height, body mass index, WC, body fat, fat free mass, fasting glucose (FG), lipid profile [triglycerides (TGs), total cholesterol (TC), HDL-c and LDL-c], physical activity level and food intake (energy, carbohydrate, total fat and protein) data were evaluated before and after the intervention.

**Results:**

Curcumin supplementation reduced body mass (p < 0.040) but did not alter other anthropometric parameters or body composition (p ≥ 0.050). In relation to the biochemical profile, the SG saw reductions in FG (p < 0.001), TGs (p < 0.001) and TC (p = 0.001) compared with the PG. At the baseline and during the intervention, the practice of physical activity and food intake did not differ between the SG and PG (p ≥ 0.050).

**Conclusion:**

Curcumin supplementation improved the blood glucose and lipid profile of Brazilian women with high WC, without altering body composition. New studies with larger sample sizes and longer durations are important for identifying more robust data regarding the proposal of this work.

## INTRODUCTION

Cardiovascular diseases (CVDs) affect heart and blood vessels and are the main cause of morbidity and mortality worldwide (1,2). About 17 million people died due to CVDs in 2015, representing 31% of all deaths globally, and more than three-quarters occurred in low- and middle-income countries (3). Among women, CVDs are more common after age 50, and the probability of death due to their occurrence is seven times higher than that of breast cancer (1,2).

High waist circumference (WC) is one of the CVD risk factors and is associated with a higher risk of all-cause mortality, independent of body mass index (BMI), and this condition can increase the risk of developing other CVD risk factors, such as type 2 diabetes mellitus and hypercholesterolemia (4–6).

Among the CVD risk factors that are considered to be modifiable, sedentary lifestyle and inadequate diet stand out (7–9). When it comes to the general population, studies have found a low adherence to a regular practice of physical exercise; a low level of physical activity at work and during leisure time; a high intake of saturated and trans fat, as well as sodium; and a low consumption of fruits, vegetables and fish, which contribute to CVD aggravation (9,10).

Thus, changes in lifestyle have been recommended as a basic strategy in the treatment of CVD risk factors (11–14). Regarding diet, studies have highlighted the presence of bioactive substances that have a cardioprotective effect, especially those that promote an improvement in blood glucose, lipid profile, blood pressure, endothelial and platelet function, and antioxidant action (15,16). In this sense, *Curcuma longa* L. (Zingiberaceae), popularly known as turmeric, which is used as a spice in many countries, including Brazil, presents a high potential in the prevention and reduction of CVD risk factors (17). Such functionality is due to the curcuminoid pigments – generically denominated curcumin, which has high antioxidant and anti-inflammatory properties attributed to the hydroxyl and methoxyl groups in the molecule (17).

Curcumin has been explored in many studies for its role in preventing obesity, possibly contributing to lipolysis and to the reduction of triglycerides and cholesterol synthesis in the liver (18,19). These actions suggest the possibility of its use as a protective factor against CVDs (17,18) and as a therapeutic intervention in the prevention of hyperglycemia (20).

Regarding the possible detrimental effect of curcumin supplementation, the literature suggests that doses of up to 12 g/day were well tolerated in humans (19). In addition, the oral bioavailability of curcumin is low due to poor gastrointestinal absorption, rapid elimination and low solubility in water (20).

The adequate dose of curcumin for the cardioprotective effect has not yet been established. Furthermore, some studies that have evaluated isolated CVD risk factors did not control for the level of physical activity and dietary intake during the intervention period (15–17,19).

Therefore, the present study was aimed at evaluating the effect of curcumin supplementation on body composition and biochemical parameters in Brazilian women with high WC.

## MATERIALS AND METHODS

### Study design, selection criteria and sample size calculation

This was a blind, randomized, placebo-controlled clinical trial conducted in 2016 with adult and elderly women in a unified health system in a Brazilian city located in the midwestern region of the country.

The basic health unit from the municipality released information about the research, and 94 women attended the meeting for the presentation of the study, but only 80 met the inclusion criteria. The inclusion criteria were: female, adult or elderly (≥20 years old), and high WC (≥80 cm) (21). Pregnant women, those allergic to dyes and those with physical and/or cognitive limitations were excluded from the study.

At the baseline and after the supplementation period, trained professionals in the city’s basic health unit collected anthropometry, body composition, physical activity level, biochemical profile and food intake data. A researcher with no clinical involvement in the trial created the stratified randomization method using Microsoft Excel^®^. This randomization method was used to eliminate potential biases in the distribution of variables that could configure confusion among groups (age, body mass, blood glucose and serum lipids). An investigator volunteer enrolled participants and randomly assigned the participants to placebo and supplemented groups (SGs) using Microsoft Excel^®^. The sample was stratified into two groups: an SG that consumed one capsule/day with 500 mg of curcumin (Curcumin C3 Complex^®^) and a placebo group (PG) that consumed one capsule/day containing 500 mg of corn starch daily for 90 days. The amount of curcumin used (500 mg/day) was based on previous studies that evaluated the effect of curcumin supplementation on body composition and glycemia (22,23). All subjects were instructed to take the supplement after lunch.

**Figure 1:**
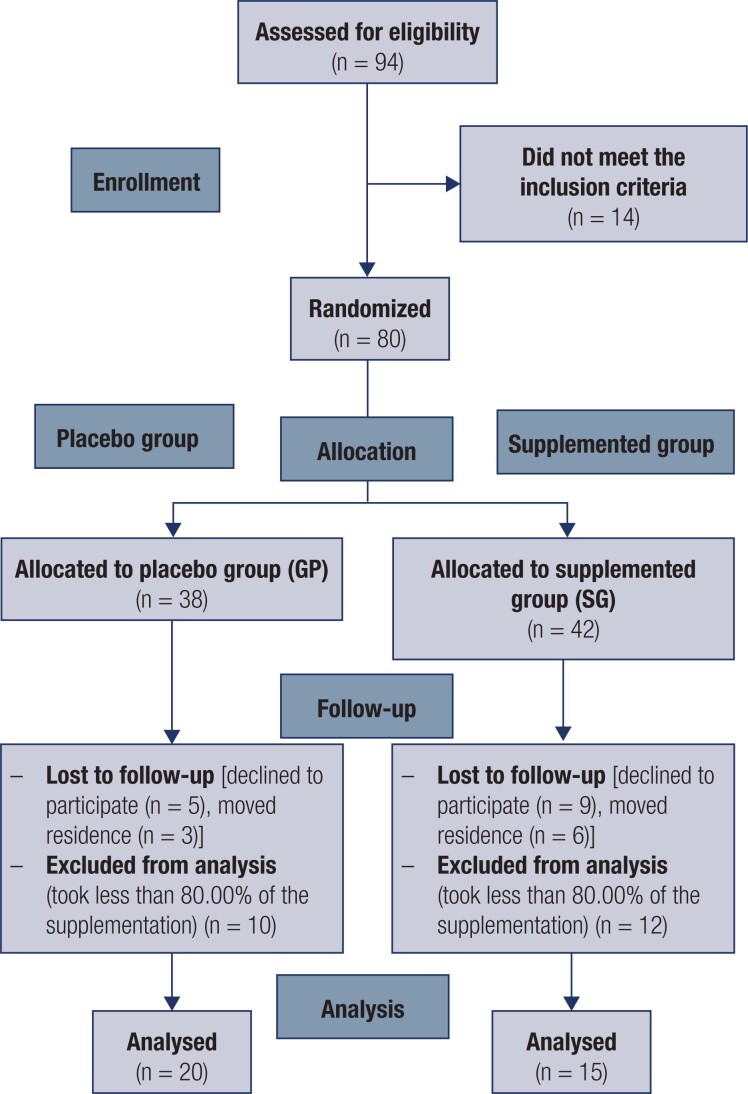
Participant flow throughout the study.

In total, 94 women were assessed for eligibility, but 14 did not meet the inclusion criteria, so 80 participants were included in the study (38 in the PG and 42 in the SG). During the follow-up, 14 subjects declined to participate, nine moved residences and 22 took less than 80.00% of the supplementation, so 35 women were included in the analysis (20 subjects in the PG were compared with 15 subjects in the SG) (Figure 1).

Regarding the sample size calculation, the sample (n = 35) had an effect size of 0.50, a power of 0.80, α = 0.05 and β = 0.20 considering “F tests: one-way fixed effects ANOVA” performed in G*Power 3.1. This effect size (0.50) means that the sample had a medium effect size (24).

### Ethical aspects

This research received approval from the Research Ethics Committee of the Federal University of Goiás, protocol nº 784.446/2014 and Certificate of Presentation for Ethical Appreciation nº 32847014.2.0000.5083. All participants were informed of the objectives and protocols of the research, and they signed the Term of Free and Informed Consent. All procedures followed the recommendations of National Health Council Resolution nº 466/2012. The present study is registered in the Brazilian Registry of Clinical Trial (ReBEC) (Universal Trial Number: U1111-1189-9960).

### Evaluation protocols

#### Anthropometry and body composition

Body mass was measured with a digital scale (Toledo^®^, São Paulo, Brazil) with a precision of 0.1 kg and a capacity of 150 kg. In addition, the height was measured with a portable stadiometer (Sanny^®^, São Paulo, Brazil). The body mass index (BMI) (kg/m^2^) was obtained.

The WC was measured at the midpoint between the lower portion of the last rib and the iliac crest with an inextensible anthropometric tape (Sanny^®^, São Paulo, Brazil) (25). The waist-to-height ratio (WHtR) was obtained by dividing the WC by the height (26).

The body composition was evaluated with a bioelectrical impedance analyzer (BIA) (RJL Systems^®^, model Quantum II, Michigan, United States of America). The resistance and reactance values were used to calculate the body fat percentage (%BF) and the fat free mass percentage (%FFM) in Body Compositions Analysis Version 2.1. All participants followed previous recommendations for BIA assessment (27).

#### Physical activity level

The physical activity level was estimated by using the International Physical Activity Questionnaire (IPAQ), which was validated for the Brazilian population. The frequency (days) and duration (minutes) of the physical activity was transformed into metabolic equivalents (METs). The sum of the METs was used to establish the level of physical activity of each participant as low, moderate or high according to the guidelines for data processing and analysis of the IPAQ short and long forms (28).

#### Biochemical parameters

Lipid profile and blood glucose were evaluated by collecting 10-mL venous blood samples. Blood serum was immediately separated via centrifugation at 25 °C and 4000 revolutions per minute (Centribio^®^, Daiki 80-2B model, Curitiba, Brazil) to determine triglycerides (TG), total cholesterol (TC), high-density lipoprotein cholesterol (HDL-c) and glucose values. All parameters were determined via enzymatic colorimetric methods with reagent kits (Doles^®^, Goiânia, Brazil), and the spectrophotometric readings were done according to the manufacturer’s instructions. The concentration of HDL-c was determined in the supernatant obtained through centrifugation after treatment with buffered polyethylene glycol (PEG 6000, Doles^®^, Goiânia, Brazil), which selectively precipitated low-density fractions [very-low-density lipoprotein cholesterol (VLDL-c) and low-density lipoprotein cholesterol (LDL-c)]. LDL-c values were estimated indirectly via a mathematical equation (29). Fasting glucose (FG) was determined via the colorimetric glucose oxidase method using reagent kits (Doles^®^, Goiânia, Brazil) following the manufacturer’s instructions. Prior to collection, participants were instructed to fast for 10-12 hours as well as to avoid alcohol consumption for three days and to maintain their usual diets the previous day.

#### Food intake estimation

The dietary intake of the participants was estimated based on six 24-hour recalls (three at the baseline and three after the intervention). Of the three questionnaires applied in each period, one was obtained on the weekend and two on a work day. The estimation of energy (kcal/day) and macronutrient (carbohydrate, total fat and protein) (g/day) intake was performed on the Diet Pro^®^ 5.0 software. For obtaining the nutritional compositions of foods, the first publication considered was the Brazilian Food Composition Table (TACO 4th edition). For foods not included in the TACO, the order of priority of publication selected was the food composition table of the Instituto Brasileiro de Geografia e Estatística (IBGE), U.S. Department of Agriculture (USDA R28) and Dietpro Table of Food Composition.

### Statistical analysis

The normality of the continuous variables was analyzed using the Shapiro-Wilk test. Parametric data were expressed as a mean ± standard deviation, and non-parametric data were expressed in the median and interquartile ranges (25^th^/75^th^ percentile).

A comparison of age, anthropometry, body composition, biochemical variables, energy expenditure in physical activity (METs) and food intake (energy and macronutrients) at the baseline between the SG and the PG was performed via the unpaired Student’s t-test with parametric variables, and via the Mann-Whitney test with non-parametric variables. The delta values of anthropometry, body composition, biochemical variables, METs and food intake were evaluated via one-way analysis of variance (ANOVA) adjusted based on the baseline values.

Double data entry was performed in Microsoft Excel^®^, and an analysis was conducted in Statistical Package Social Science 21.0. P < 0.050 was considered to be significant.

## RESULTS

In relation to age, anthropometry, body composition, biochemical parameters, METs and food intake, only the baseline value of height was different between the groups (1.59 ± 0.06 m in SG *vs.* 1.54 ± 0.07 m in PG) (p = 0.005) (Table 1).

**Table 1:** Age, anthropometry, body composition, biochemical parameters and energy expenditure in physical activity and food intake at baseline of Brazilian women with high waist circumference

	Supplemented group (n = 20)	Placebo group (n = 15)	P value
Age (years)	47.00 (10.52)	50.00 (12.58)	0.242^1^
Body mass (kg)	78.97 (16.03)	74.54 (12.85)	0.387^1^
Height (m)	1.59 (0.06)	1.54 (0.07)	0.005^1^
BMI (kg/m^2^)	31.11 (5.98)	31.08 (4.99)	0.988^1^
WC (cm)	97.67 (12.59)	94.83 (7.65)	0.446^1^
WHtR	0.61 (0.08)	0.61 (0.06)	0.990^1^
BF (%)	36.60 (5.87)	35.82 (3.95)	0.660^1^
FFM (%)	61.82 (5.93)	63.52 (5.83)	0.406^1^
FG (mg/dL)	88.75 (13.92)	89.00 (74.94/99.99)	0.689^2^
TG (mg/dL)	154.00 (139.79/176.01)	151.93 (32.93)	0.714^2^
TC (mg/dL)	191.50 (172.76/198.84)	194.73 (28.35)	0.894^2^
HDL-c (mg/dL)	55.10 (9.88)	52.60 (11.13)	0.488^1^
LDL-c (mg/dL)	97.08 (30.87)	111.75 (30.63)	0.172^1^
METs	646.24 (291.75)	580.50 (177.95)	0.414^1^
Energy intake (kcal)	1637.65 (1468.89/1750.35)	1654.83 (179.90)	0.571^2^
Carbohydrate (g)	218.56 (48.77)	244.45 (36.63)	0.095^1^
Total fat (g)	48.62 (38.87/113.34)	47.18 (10.37)	0.739^2^
Protein (g)	71.48 (12.50)	63.10 (14.21)	0.073^1^

BMI: body mass index; WC: waist circumference; WHtR: waist-to-height ratio; BF: body fat; FFM: fat free mass; FG: fasting glucose; TG: triglycerides; TC: total cholesterol; HDL-c: high-density lipoprotein cholesterol; LDL-c: low-density lipoprotein cholesterol; METs: metabolic equivalents.

Parametric data: expressed as mean ± standard deviation. Non-parametric data: expressed as median and interquartile ranges (25^th^/75^th^ percentile).

1 Unpaired Student’s t-test.

2 Mann-Whitney test.

After the intervention, the SG witnessed a reduction in body mass (p = 0.040) compared with the PG, with no difference being found in BMI, WC, WHtR, %BF and %FFM (p ≥ 0.050) (Table 2). Regarding the biochemical parameters, curcumin supplementation reduced FG (p < 0.001), TG (p < 0.001) and TC (p = 0.001) in the SG compared with the PG, with no difference being found in HDL-c (p = 0.683) and LDL-c (p = 0.561) (Table 3). No side or adverse effects were reported during the supplementation period.

**Table 2:** Anthropometric and body composition parameters of Brazilian women with high waist circumference

Variables	Supplemented group (n = 20)			Placebo group (n = 15)			P value^1^
	Baseline	90 days	Delta	Baseline	90 days	Delta	
Body mass (kg)	78.97 (16.03)	77.04 (15.56)	-1.25 (-2.66/-1.18)	74.54 (12.85)	73.63 (12.36)	-0.50 (-1.96/0.13)	0.040
BMI (kg/m^2^)	31.11 (5.98)	30.35 (5.81)	-0.47 (-1.00/-0.46)	31.08 (4.99)	30.70 (4.81)	-0.17 (-0.83/0.07)	0.070
WC (cm)	97.67 (12.59)	96.85 (14.99)	-2.00 (-4.72/2.84)	94.83 (7.65)	93.67 (6.90)	-1.16 (3.50)	0.140
WHtR	0.61 (0.08)	0.61 (0.09)	-0.01 (-0.03/0.01)	0.61 (0.06)	0.60 (0.05)	-0.01 (0.02)	0.343
BF (%)	36.60 (5.87)	34.52 (5.98)	-2.08 (2.52)	35.82 (3.95)	34.95 (3.35)	-0.86 (1.63)	0.093
FFM (%)	61.82 (5.93)	65.48 (5.98)	3.65 (8.51)	63.52 (5.83)	65.04 (3.35)	1.52 (5.91)	0.413

BMI: body mass index; WC: waist circumference; WHtR: waist-to-height ratio; BF: body fat; FFM: fat free mass.

Parametric data: expressed as mean ± standard deviation. Non-parametric data: expressed as median and interquartile range (25^th^/75^th^ percentile).

1 One-way ANOVA (adjusted by baseline values) between-group comparisons of delta values.

**Table 3:** Biochemical parameters of Brazilian women with high waist circumference

Variables	Supplemented group (n = 20)			Placebo group (n = 15)			P value^1^
	Baseline	90 days	Delta	Baseline	90 days	Delta	
FG (mg/dL)	88.75 (13.92)	80.80 (11.10)	-6.70 (13.00)	89.00 (74.94/99.99)	94.00 (84.99/ 110.08)	6.33 (19.66)	<0.001
TG (mg/dL)	154.00 (139.79/176.01)	144.00 (133.09/147.61)	-17.55 (37.35)	151.93 (32.93)	140.35 (15.51)	19.27 (22.84)	<0.001
TC (mg/dL)	191.50 (172.76/198.84)	170.55 (160.46/180.64)	-15.25 (19.22)	194.73 (28.35)	191.00 (184.36/ 218.31)	6.60 (14.00)	0.001
HDL-c (mg/dL)	55.10 (9.88)	59.55 (7.80)	4.45 (9.00)	52.60 (11.13)	49.00 (47.67/ 63.40)	-2.93 (12.81)	0.683
LDL-c (mg/dL)	97.08 (30.87)	83.43 (22.02)	-13.65 (19.74)	111.75 (30.63)	107.23 (37.53)	-4.52 (30.84)	0.561

FG: fasting glucose; TG: triglycerides; TC: total cholesterol; HDL-c: high-density lipoprotein cholesterol; LDL-c: low-density lipoprotein cholesterol.

Parametric data: expressed as mean ± standard deviation. Non-parametric data: expressed as median and interquartile ranges (25^th^/75^th^ percentile).

1 One-way ANOVA (adjusted by baseline values) between groups comparisons of delta values.

No difference was found in METs (p = 0.258), energy intake (p = 0.146), carbohydrate (p = 0.103), total fat (p = 0.156) and protein (p = 0.160) intake between groups during the intervention (Table 4).

**Table 4:** Energy expenditure in physical activity and energy and macronutrient intake of Brazilian women with high waist circumference.

Variables	Supplemented group (n = 20)			Placebo group (n = 15)			P value^1^
	Baseline	90 days	Delta	Baseline	90 days	Delta	
METs	646.24 (291.75)	636.00 (198.00/1272.00)	-16.88 (88.63)	580.50 (177.95)	559.10 (138.36)	-21.40 (129.34)	0.258
Energy intake (kcal)	1637.65 (1468.89/1750.35)	1633.20 (232.69)	-4.44 (138.16)	1654.83 (179.90)	1658.74 (212.04)	3.92 (-69.99/52.77)	0.146
Carbohydrate (g)	218.56 (48.77)	233.65 (37.88)	7.72 (30.81)	244.45 (36.63)	243.47 (38.51)	-0.98 (-23.84/1.48)	0.103
Total fat (g)	48.62 (38.87/113.34)	47.55 (27.74/101.65)	-4.08 (-5.13/1.54)	47.18 (10.37)	47.81 (10.45)	0.63 (4.52)	0.156
Protein (g)	71.48 (12.50)	71.83 (11.26)	0.36 (5.43)	63.10 (14.21)	63.63 (14.04)	0.53 (3.10)	0.160

METs: metabolic equivalents.

Parametric data: expressed as mean ± standard deviation. Non-parametric data: expressed as median and interquartile ranges (25^th^/75^th^ percentile).

1 One-way ANOVA (adjusted by baseline values) between-group comparisons of delta values.

## DISCUSSION

This study demonstrated that 500 mg/day of curcumin for 90 days had beneficial effects on the health of women with high WC, with reductions being found in FG, TG and TC compared with the PG.

The effects of curcumin supplementation on CVD risk factors were evaluated in individuals of different ages as well as physiological and pathological conditions. An investigation compared the effects of curcumin supplementation at different doses (500 mg/day and 6 g/day) on healthy subjects’ lipid profile and found that the first dose (500 mg/day) was more effective in reducing serum TC and TG (30).

DiSilvestro and cols. (31) supplemented healthy men and women, aged 40-60 years, with 80 mg/day of curcumin in lipid form for four weeks. The authors found a reduction in TG concentration (p < 0.050), which was attributed to the absorptive capacity of the lipid form of curcumin, but they did not observe a change in TC (p ≥ 0.050), which may be due to the low concentration of the supplement.

The lipid-lowering effect of curcumin was also evaluated in a crossover study with 30 obese subjects who consumed 1g/day of the supplement or a placebo for one month, with a reduction in TG (p = 0.009) being found in the SG (32).

A study with 117 subjects with metabolic syndrome identified that those who received 1g/day of curcumin for eight weeks experienced reductions not only in LDL-c, TC and FG but also in pro-inflammatory markers, such as tumor necrosis factor alpha (TNF)-α, interleukin (IL)-6, transforming growth factor (TGF)-β and monocyte chemoattractant protein (MCP)-1 (p < 0.001) when compared with the PG (33).

The consumption of 500 mg/day of curcumin for eight weeks managed to reduce BMI, WC, serum TC, LDL-c and TG (p < 0.050) in 87 adults, both genders, with overweight and fatty liver disease (34).

The effect of curcumin supplementation alone and combined with phytosterol for four weeks was evaluated in adults, both genders, with high WC, overweight and hypercholesterolemia. The results showed that curcumin potentialized the effects of phytosterol on the modulation of lipid parameters and highlighted the need for further studies on the mechanisms of action of curcumin to confirm its cardioprotective effect. As in the present study, a reduction in FG was found, but this change was not clarified, as it occurred only in the group that received curcumin alone (35).

Some possible mechanisms of action of curcumin on the improvement of blood glucose are the inhibition of 11β-hydroxysteroid dehydrogenase 1 (11β-HSD1), the enhancement of insulin secretion from pancreatic cells by increasing the stimulation of glucagon-like peptide-1 secretion, and the reduction of hepatic glucose production (36–38). In relation to the improvement of lipid profile, curcumin seems to increase the activity of cholesterol-7ɑ-hydroxylase and fatty acid β-oxidation, as well as reduce the absorption of cholesterol in the gut, serum free fatty acid and the expression of lipogenic genes (34,39–41). Curcumin also improves insulin secretion and lipid metabolism by activating peroxisome proliferator-activated receptor (PPAR)-γ (42).

Despite the reduction of body mass in the SG compared with the PG (p = 0.040), curcumin supplementation did not reduce body fat or increase lean body mass, as no difference was found in %BF and %FFM between the groups (p = 0.093 and 0.413, respectively). Thus, curcumin supplementation did not improve body composition. One hypothesis was the loss of total body water in the SG compared with the PG, a parameter that was not evaluated, which was a limitation of this study. Other limitations of the study included the limited number of participants and the method of body composition assessment used (BIA), as the accuracy of the result depends on the hydration status of the individual. However, the strengths of this study were the length of the intervention, the discussion of a non-pharmacological approach for the treatment of obesity-related comorbidities and the use of a bioactive compound derived from a spice grown and widely used in the region in which the study was conducted.

In this study, 12 participants in the SG and 10 in the PG were excluded from analysis for taking less than 80.00% of the supplementation. Among the factors that may have contributed to this are forgetting to take the capsule, a long course of treatment and mistrust of the supplement’s benefits (43).

Finally, it is worth mentioning that intention-to-treat analysis was not performed because in the present study, the treatment was effective, and the nonadherence was substantial. In this case, this analysis could underestimate the magnitude of the treatment effect that occurred in adherent patients (44).

In conclusion, the results found in the present study showed that curcumin supplementation improved the blood glucose and lipid profile of Brazilian women with high WC, without a difference in body composition. In this sense, the use of curcumin would be effective for preventing increases in these cardiometabolic parameters in women who already have CVD risk factors. This would help them to avoid the deterioration of the health status of this population.

However, further studies are needed to determine the adequate dose and period of treatment for the prevention of the CVD risk factors.
